# Meal-time Smartphone Use in an Obesogenic Environment: Two Longitudinal Observational Studies

**DOI:** 10.2196/22929

**Published:** 2021-05-06

**Authors:** Joceline Y Y Yong, Eddie M W Tong, Jean C J Liu

**Affiliations:** 1 Division of Social Sciences Yale–National University of Singapore College Singapore Singapore; 2 Department of Psychology National University of Singapore Singapore Singapore; 3 Neuroscience and Behavioral Disorders Programme Duke-National University of Singapore Medical School Singapore Singapore

**Keywords:** screen time, mobile phones, technology, obesogenic environment, young adults

## Abstract

**Background:**

Despite a large volume of research on the impact of other digital screens (eg, televisions) on eating behavior, little is known about the nature and impact of mealtime smartphone use.

**Objective:**

We investigated how smartphones are used in everyday meals, whether phone users differ according to mealtime phone use patterns, and whether specific phone functions (particularly food photography) would affect the amount and enjoyment of food eaten.

**Methods:**

Across 2 studies, we used the experience sampling method to track 1780 meals in situ. In study 1, a total 137 young adults reported on their mealtime smartphone use 3 times per day over 7 consecutive days. This corresponded to each main meal, with participants recording whether they used their phones and what phone functions they engaged in while eating. In study 2, a total of 71 young adults were similarly tracked for 3 meals per day over 7 days. Across the week, participants’ meals were randomized to 1 of 3 smartphone conditions: food photography while eating, nonfood photography while eating, or no phone use. As the outcome measures, participants reported on the amount and enjoyment of food they ate.

**Results:**

During the week-long tracking, most participants (110/129, 85.3%) recorded at least one instance of mealtime smartphone use, with an average frequency of 1 in 3 meals where phones were used (27.1%; 95% CI 23.6-30.6). Unlike traditional digital screens, mealtime phone use encompassed a wide range of social and nonsocial activities. Further, specific forms of phone use behaviors influenced food intake in different ways. Specifically, in study 2, participants showed the typical pattern of increased food intake across the day when they engaged in nonfood photography during a meal (*P*<.001); however, this pattern was disrupted when they engaged in food photography (*P*=.73).

**Conclusions:**

Our findings underscore the prevalence and multifaceted nature of mealtime phone use, distinguishing mobile phones from traditional forms of digital screens.

**Trial Registration:**

ClinicalTrials.gov NCT03299075; https://www.clinicaltrials.gov/ct2/show/NCT03299075 and ClinicalTrials.gov NCT03346785; https://clinicaltrials.gov/ct2/show/NCT03346785

## Introduction

A large body of research underscores how using digital screens (eg, television, computers) can predispose an individual to obesity [1-3]. For example, epidemiological studies consistently report that increased screen time is associated with decreased physical activity and a higher BMI [1-6]. In laboratory studies, those who watch television or play computer games while eating were found to consume more calories [7-9], be less aware of how much food they have eaten [9,10], and rely more heavily on external cues to determine satiety [11]. Accordingly, various regulatory and industry groups (eg, the American Academy of Pediatrics) have issued guidelines advocating for digital screens to be put aside during meal times [12-14].

Although these guidelines have been based primarily on television research, mobile phones (or smartphones) have also been linked to weight gain [15,16] and are increasingly being used at the dinner table [17-19]. This is unsurprising given that smartphone penetration rates have risen globally [20], particularly among younger individuals (aged 18-34 years), who report greater use of smartphones, the internet, and social media (relative to older adults aged over 50 years) [21]. Among youths, the rate of smartphone ownership reaches 84%-99% in countries with advanced economies [21].

Despite the ubiquity of mobile devices, there have been few studies on mealtime phone use, and the impact of this phenomenon remains unclear. On the one hand, a mobile phone resembles other digital screens in its sedentary usage [22,23] and ability to distract the phone user [24,25]. On the other hand, the vast number of smartphone functions may mean that someone using a phone while eating may engage with food in ways that differ from traditional digital screens [26].

Of note, one particular form of mealtime phone use has received heavy criticism, that of food photography, where the meal is photographed and the images are shared on social media. Although the capacity to take photographs pre-dates mobile phones, incorporating camera functions into the phone has transformed the extent and means by which we capture our meals. Correspondingly, food pictures now rank among the top categories of images uploaded on the photo-sharing platform Instagram.

Food photography is illustrative of how mealtime phone use can introduce new rituals to the dinner table. Unlike other forms of digital screens (or indeed other forms of phone use), taking photographs involves direct engagement with the meal—arranging the food, taking a photograph, applying a filter, and posting the photo on social media. In the public domain, restaurateurs have been so concerned that this would detract from the eating experience that they moved to ban it [27]. Similarly, this act was singled out in clinical circles as being potentially pathological [28]. Together, these concerns point to a broader conversation on how advances in mobile phone technology have the potential to transform eating habits.

In light of these developments, there is a need for empirical research to understand the place of phones at the dinner table and how they contribute to an obesogenic environment [29]. To this end, we conducted two 2 studies (NCT03299075 and NCT03346785) using the experience sampling method to capture mealtime phone use as it occurred in day-to-day routines (a method frequently applied in health psychology research [30-32]). By tracking usage patterns in real time, this method had high ecological validity and circumvented recollection biases associated with traditional surveys [33]. This is particularly important when studying phone use, as users have been found to provide poor self-reported estimates on how frequently and in what context they use their phones [34]. Using experience sampling, we thus investigated how mobile phones are used in everyday meals (study 1), whether the characteristics of phone users differ according to mealtime phone use patterns (study 1), and how specific phone functions, particularly food photography, may affect the amount and enjoyment of food eaten (study 2).

## Methods

### Recruitment

Across both studies, participants were recruited from the National University of Singapore via advertisements. All participants provided informed consent at the start of the study and were reimbursed SGD $5 (study 1) or SGD $10 (study 2) for their time. Protocols were approved by the National University of Singapore’s Institutional Review Board (A-15-170) and were preregistered at ClinicalTrials.gov (NCT03299075 and NCT03346785).

### Participants

In the first study, participants were 137 young adults who met the following eligibility criteria: ownership of a smartphone, aged between 18-30 years, with no history of medical or psychiatric disorders (including eating disorders), and nonsmoker status. The second study involved 71 young adults recruited using the same eligibility criteria as study 1. (For both studies, Multimedia Appendix 1 provides a detailed flow diagram of participant selection and dropout.)

### Procedures

As baseline measures, participants self-reported demographic information (age, gender, ethnicity). They also reported 3 health-related metrics: their height and weight (used to derive their BMI), and their frequency of engaging in vigorous physical activity (defined as the number of times, during a single week, they engaged in activities where they “worked up a sweat”).

Participants also completed the Dutch Eating Behavior Questionnaire (DEBQ) [35], a 33-item scale that determined whether participants deliberately restrained their eating (eg, by refusing food or drink because of weight concerns), ate in response to emotions (eg, when irritated), or ate in response to the external environment (eg, when food smelled and looked good). Finally, participants reported on their habitual phone use patterns [26] (ie, contexts during which participants used their phones, phone functions used, and social media activity).

For the experience sampling component, participants were contacted daily for 7 days (Monday to Sunday) via the Facebook Messenger app (Facebook, Inc) on their mobile phones. Each day, participants received 3 prompts sent at customized schedules coinciding with their regular meal times (breakfast, lunch, and dinner). These prompts were delivered at the following median times: 9:15 AM (study 1) and 8:45 AM (study 2) for breakfast, 12:45 PM (studies 1 and 2) for lunch, and 6:45 PM (studies 1 and 2) for dinner

All messages were delivered via a Python-programmed Facebook bot, and responses were recorded through the Messenger platform with a 30-minute time-out window (median response rate for study 1: 16/21,76%; for study 2: 21/21, 100%). A full list of questions asked of participants can be found in Multimedia Appendix 2.

### Experience Sampling: Questions on Mealtime Phone Use (Study 1)

As study 1 was designed to understand everyday mealtime phone use, participants were asked at each prompt whether they had eaten in the last 15 minutes. In this manner, we captured 1140 meals within the day-to-day routines of 129 free-living participants.

If participants reported that they had eaten, they then indicated whether they used their phones during the meal and what phone functions they used. As a pilot for study 2, participants also recorded their consumption patterns at each prompt (Multimedia Appendix 3).

### Experience Sampling: Experiment on the Impact of Phone Use Patterns (Study 2)

In study 2, we sought to investigate the causal impact of phone use, particularly that of mealtime food photography, on eating behaviors. As food photography involves a set of rituals in naturalistic settings (eg, arranging the food, taking photos from different angles, applying photo filters) [36], we again used experience sampling to study this phenomenon in situ. To this end, we first asked participants at each prompt if they were going to eat within the next 15 minutes, allowing us to capture 640 meals from 70 participants.

To allow causal inferences, we then randomly assigned meals to 1 of 3 phone use conditions. If participants had indicated that they were about to eat, they were sent one of the following instructions: (1) take a photograph of their food as if they were doing so for the photo-sharing application Instagram (food photography condition), (2) take a photograph of their surroundings (eg, furniture, decorations) as if for Instagram (matched nonfood photography condition), (3) or refrain from phone use (no phone condition). These instructions were randomized within the week, with each participant experiencing all 3 conditions. Critically, a follow-up message was then sent 30 minutes after the instructions to verify compliance: participants were either asked to upload the photograph they took (for the food and nonfood photography conditions; see Multimedia Appendix 4 for sample images) or to indicate whether they had used their phones while eating (for the no phone condition).

Finally, as the primary outcome measures, we tracked participants’ enjoyment of the meal and the amount they had eaten. At the end of each prompt, participants rated how much they enjoyed the food using a 7-point scale anchored with “1 = not at all” and “7 = very much”. To assess the amount eaten, we took a relative measurement approach based on food consumption studies that had employed experience sampling [37]. Using a 7-point scale anchored with “1 = less than normal” and “7 = more than normal,” participants rated how much they had eaten. Self-reported portions increased across the day (from breakfast to lunch to dinner; main effect of meal type on amount: *t*_276,66_=3.12; *P*=.002), mirroring the diurnal intake rhythms that have been observed in food diary studies of free-living individuals [38-40] and thus validating this approach (see Multimedia Appendix 3 for validation analyses).

### Statistical Analyses

To characterize mealtime phone use patterns (study 1), observations are summarized with counts and percentages. Characteristics of phone users themselves are summarized with means (SD) or counts and percentages, with subgroups of phone users compared using *t* tests.

For Study 2, we analyzed all instances where participants complied to the experimental instructions. Following the analysis strategy of screen-time studies, we planned a set of orthogonal contrasts to first assess the impact of phone use (vs no phone use) [9] and the impact of different forms of phone use (food vs nonfood photography) [41]. Applying contrast coding, we coded whether participants had been assigned to use their phones during the meal (phone use variable: –1=no; 0.5=yes, food photography; 0.5=yes, nonfood photography), and, if they had, we coded what form of phone photography they had engaged in (photography variable: 0=no phone; –1=nonfood photography; 1=food photography). For each outcome measure (enjoyment and amount), we ran a linear mixed-effects model with time (centered and divided by 3 to put the unit in days), phone use, photography, and meal type (breakfast, lunch, or dinner), with the interaction between each phone variable and meal type entered as fixed effects. Random intercepts accounted for correlated data due to repeated measures, and the type 1 decisionwise error rate was controlled at α=.05.

Across both studies, analyses were conducted using SPSS 25 (IBM Corp) and R 3.4.0 (R Foundation for Statistical Computing).

## Results

### Baseline Participant Characteristics

In study 1, the participants had a mean age of 21.68 years (SD 2.07), a mean BMI of 20.89 (SD 2.80), and were predominantly of Asian ethnicity: 85.3%(110/129) self-identified as Chinese, 3.8% (5/129) Indian, 3.1% (4/129) Malay, 1.6% (2/129) Eurasian, and 6.2% (8/129) as another ethnicity. Moreover, 73.6% (95/129) identified as female. In study 2, the participants had a mean age of 22.29 years (SD 2.36), a mean BMI of 21.80 (SD 3.22), and were predominantly of Asian ethnicity: 89% (62/70) self-identified as Chinese, 4% (3/70) Indian, 1% (1/70) Malay, and 6% (4/70) another ethnicity. Moreover, 57 of the 70 participants (81%) identified as female.

Across both studies, the average participant’s BMI fell within the normal range [42]. Relative to the resident population, both samples had a higher proportion of females and persons of Chinese ethnicity (>10% difference) [43].

### How Mobile Phones Are Used in Everyday Meals (Study 1)

**Figure 1:**
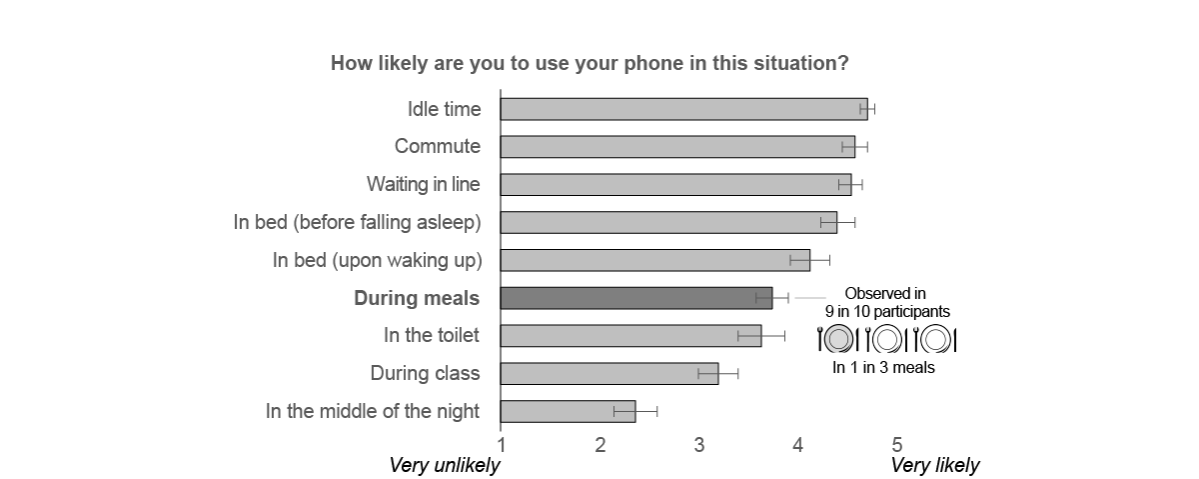
In a baseline questionnaire on habitual phone use, participants reported how likely they were to use their phones in each context. A higher score corresponds to greater likelihood, and horizontal lines represent the 95% CI for the mean. When participants were then monitored for 1 week, mealtime phone use was observed in approximately 9 of 10 participants (in an average of 1 in 3 meals).

In the baseline phone-use questionnaire, participants self-reported that they were “likely” to use their phones during a meal (mean rating 3.74/5; 95% CI 3.58-3.91; see Figure 1). This rating was quantified by the experience sampling data: during the week of in-depth monitoring, the vast majority of participants (110/129, 85.3%) recorded at least one instance of mealtime phone use. On average, participants used their phones during 1 of 3 meals (27.1%; 95% CI 23.6-30.6).

**Figure 2:**
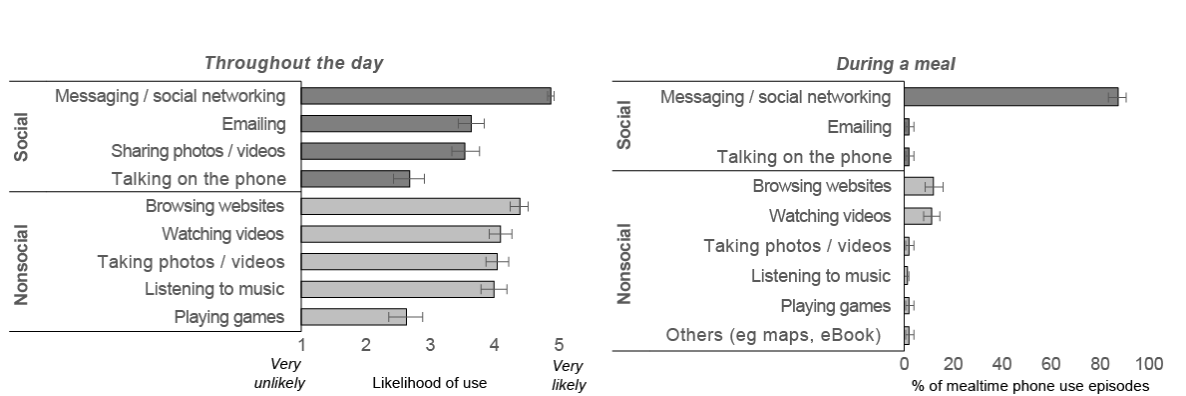
(Left) In a baseline questionnaire on habitual phone use, participants reported how likely they were to use each phone function on a regular day. A higher score corresponds to greater likelihood, and horizontal lines represent the 95% CI for the mean. (Right) Participants were then monitored closely for 1 week; the graph on the right depicts the percent of mealtime phone use episodes where each phone activity was recorded. Horizontal lines represent the 95% CI for each percentage.

In terms of how phones were used, our week-long monitoring captured a wide range of social (eg, talking on the phone) and nonsocial phone activities (eg, listening to music) that participants engaged in while eating. These patterns broadly mapped onto those outside the eating context (as reported in previous survey studies [26,44,45] and by participants in the baseline questionnaires; Figure 2). Namely, across all contexts, a similar variety and rank ordering of phone functions were used (Kendall τ=0.64; *P*=.03).

However, mealtime phone patterns were distinct in one important aspect. Although messaging or social networking was the top-ranked functions across all contexts, participants were far more likely to use this feature while eating than to use any other phone function. Indeed, 86.5% (313/362; 95% CI 83.5-90.4) of all mealtime phone use episodes captured involved messaging or social networking, which was 8 times the frequency of the next most widely used function, browsing websites (39/362, 11%; 95% CI 8.7-15.3). Correspondingly, mealtime phone use was more likely to be social than nonsocial in nature. Outside the eating context, however, participants self-reported that they used a range of social and nonsocial phone functions comparably (mean rating of 4.0-4.88 for the likelihood of listening to music, taking photos or videos, watching videos, browsing websites, and messaging or social networking).

### Characteristics of Phone Users as a Function of Mealtime Usage (Study 1)

**Figure 3:**
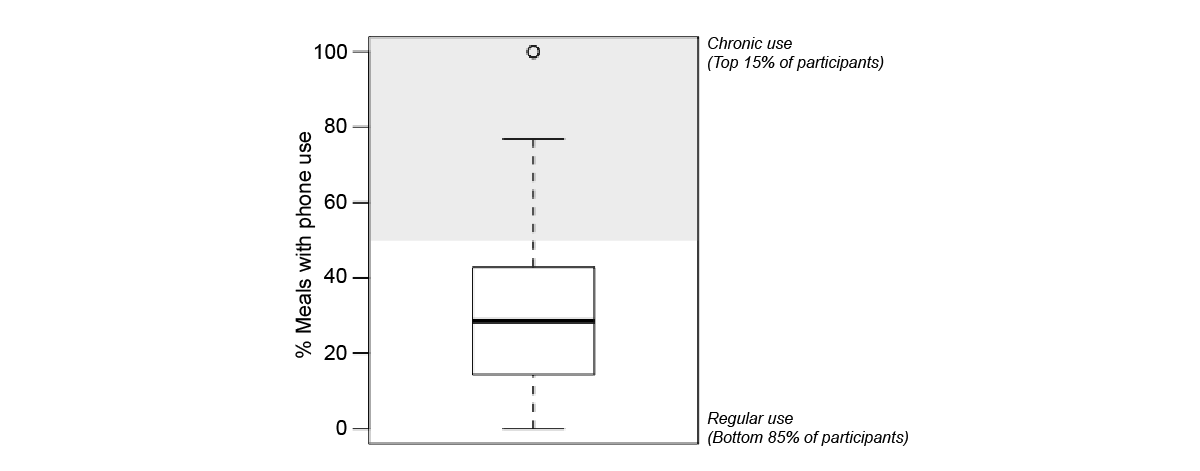
Box-plot depicting the distribution of mealtime phone use frequency captured across 1 week of naturalistic monitoring. The bottom, midline, and top of the box represent the 25th, 50th, and 75th percentiles, respectively, and chronic users are represented in the shaded gray area (top 15% of participants, corresponding to ≥50% of meals with phone use).

The experience sampling method captured large individual differences in mealtime phone use patterns. Across the week of observation, we recorded the full range of 0%-100% of meals where phones were used (Figure 3; Multimedia Appendix 5 shows how this was not merely an artifact of the number of meals captured per participant). Accordingly, we conducted exploratory subgroup analyses to examine whether “chronic” mealtime phone users (the top 15% of participants in phone usage, corresponding to 50%-100% of meals with phone use) differed in characteristics from “regular” mealtime phone users (the remaining 85% of participants, corresponding to <50% of meals with phone use).

As shown in Table 1, chronic mealtime phone users tended to be older (*t*_127_=–1.94; *P*=.05) and to use their phones more frequently for watching videos (*t*_127_=–2.28; *P*=.02). However, we found no evidence that chronic users differed in BMI, levels of vigorous physical activity each week, or habitual eating patterns as measured by the DEBQ (smallest *P*=.19).

**Table 1:** Participant characteristics as a function of mealtime phone use patterns^a^.

Characteristic				Chronic mealtime phone users (n=22)	Regular mealtime phone users (n=107)	Test statistic (*P* value)			
**Observed mealtime phone use**									
	Proportion of meals with phone use			59.83 (11.77)	20.31 (13.73)	–12.58 (*<.001*)^b^			
**Demographics^c^**									
	Age (years)			22.45 (2.44)	21.52 (1.96)	–1.94 (.05)			
	**Gender, n (%)**						0.18 (.67)^d^		
			Female	17 (77.27)	78 (72.90)				
			Male	5 (22.73)	29 (27.10)				
	**Ethnicity, n (%)**						2.51 (.64)^d^		
			Chinese	19 (86.36)	91 (85.05)				
			Malay	0 (0.00)	4 (3.74)				
			Indian	1 (4.55)	4 (3.74)				
			Eurasian	1 (4.55)	1 (0.93)				
			Others	1 (4.55)	7 (6.54)				
	**Phone brand, n (%)**						2.63 (.62)^d^		
			Apple	15 (68.18)	74 (69.16)				
			Samsung	4 (18.18)	19 (17.76)				
			Others	3 (13.64)	14 (13.08)				
**Health-related variables^c^**									
	BMI			20.18 (2.27)	21.04 (2.88)	1.31 (.19)			
	**Frequency of vigorous physical activity, n (%)**						0.30 (.86)^d^		
		0-2 times/week		15 (0.68)	68 (63.55)				
		3-4 times/week		6 (27.27)	31 (28.97)				
		5-7 times/week		1 (4.55)	8 (7.48)				
**Dutch Eating Behavior Questionnaire^c^**									
	Restrained eating score			2.30 (0.66)	2.48 (0.83)	0.95 (.34)			
	Emotional eating score			2.50 (0.96)	2.61 (0.80)	0.56 (.58)			
	External eating score			3.39 (0.62)	3.32 (0.54)	0.57 (.57)			
**Self-reported phone use habits^c^**									
	**Social phone functions engaged in**								
		Messaging or social networking		4.86 (0.35)	4.89 (0.32)	0.32 (.75)			
		Emailing		3.82 (1.05)	3.60 (1.12)	–0.85 (.40)			
		Sharing photos or videos		3.50 (1.34)	3.56 (1.15)	–0.19 (.85)			
		Talking on the phone		2.73 (1.32)	2.65 (1.30)	–0.24 (.81)			
	**Nonsocial phone functions engaged in**								
		Browsing websites		4.50 (0.60)	4.36 (0.79)	–0.76 (.45)			
		Watching videos		4.55 (0.51)	4.01 (1.08)	2.28 (*.02*)			
		Taking photos / videos		3.91 (1.07)	4.07 (0.96)	0.68 (.50)			
		Listening to music		3.82 (1.14)	4.04 (1.13)	0.83 (.41)			
		Playing games		2.82 (1.76)	2.58 (1.43)	0.80 (.43)			
	**Typical context for phone use**								
		Idle time		4.82 (0.40)	4.67 (0.47)	–1.35 (.18)			
		During commute		4.73 (0.55)	4.55 (0.76)	–1.04 (.30)			
		While waiting in line		4.64 (0.49)	4.50 (0.69)	–0.85 (.40)			
		In bed before falling asleep		4.64 (0.73)	4.35 (0.98)	–1.31 (.20)			
		In bed when waking up		4.23 (1.02)	4.09 (1.15)	–0.51 (.62)			
		During meals		4.09 (0.61)	3.67 (1.00)	–1.89 (.06)			
		In the toilet		3.45 (1.57)	3.66 (1.30)	0.66 (.51)			
		During class time		3.32 (1.29)	3.18 (1.11)	–0.53 (.60)			
		In the middle of the night		2.55 (1.54)	2.31 (1.22)	–0.79 (.43)			
	**Social networking involvement**								
		Number of Instagram followers		413.0 (243.81)	534.3 (735.23)	0.71 (.48)			
		Number of Instagram accounts followed		598.3 (471.74)	487.1 (261.84)	–1.45 (.15)			

^a^Unless otherwise stated, the data are reported as means (SD), and the test statistic refers to the *t* statistic.

^b^Italics indicate *P* value <.05.

^c^Based on responses to the baseline questionnaires.

^d^Chi-square statistic reported.

### Impact of Mealtime Phone Use Patterns on Eating Behaviors (Study 2)

**Figure 4:**
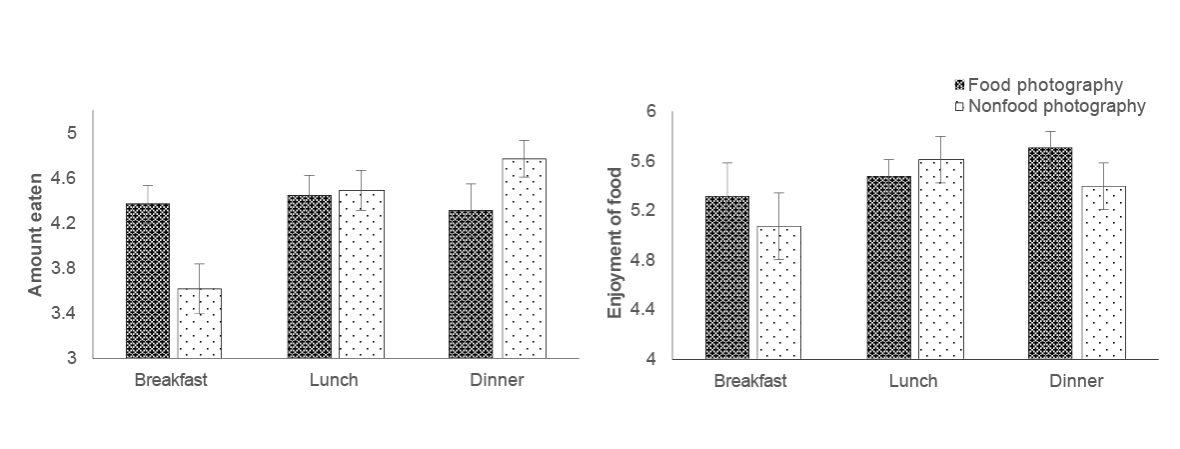
Mean amount and enjoyment of food eaten at each meal (breakfast, lunch, or dinner), plotted as a function of whether participants engaged in food photography or nonfood photography. A higher score corresponds to greater enjoyment or amount eaten, and vertical lines represent 1 SE of the mean.

In Study 2, we used linear mixed-effects models to examine the causal impact of phone use patterns, particularly mealtime food photography, on the amount and enjoyment of food eaten. For food intake, we observed a significant interaction between photography condition and meal type (*t*_324,60_; =–2.61; *P*=.009). As shown in Figure 4, when participants photographed nonfood items during a meal, they showed the typical increase of food intake from breakfast to lunch and dinner (effect of meal for nonfood condition: *t*_93,81_=4.15; *P*<.001). On the other hand, when they took mealtime photographs of their food, there was no significant effect of the type of meal consumed (effect of meal for food condition: *t*_106,34_=0.35; *P*=.73). In other words, when participants took photographs of their food, we found no evidence that they discriminated between breakfast, lunch, or dinner in the amount of food eaten. Finally, there was no significant main or interaction effect involving phone use more generally (that is, whether or not participants used their phones during the meal; smallest *P*=.25).

In terms of the enjoyment of food eaten, there were no significant effects involving photography condition (smallest *P*=.72) or general phone use (smallest *P*=.48; Figure 4; for the 2 mixed-effects models, detailed model output including parameter estimates can be found in Multimedia Appendix 6.)

## Discussion

### Principal Findings

In this series of studies, we documented how mobile phones have found a place at the modern-day dining table. During 1 week of in-depth monitoring, nearly 9 in 10 of our participants recorded at least one instance of mealtime phone use. This widespread prevalence points to new norms in eating habits, giving impetus for research on the phenomenon.

As the first step in this direction, we recorded what activities participants engaged in when they used their phones during a meal. As compared to traditional digital screens where participants perform only one function (eg, watching television), mealtime phone use entails a wide range of activities that are both social (eg, sending messages to peers) and nonsocial (eg, watching videos) in nature. This has implications for clinical guidelines and research, both of which have classified phone use as “screen time” without differentiating mobile phones from other forms of digital screens, or between various mobile phone activities [12-14]. By contrast, our findings suggest a need to reframe mealtime phone use. Given the wide range of activities engaged in, phone use may be more profitably viewed as multidimensional rather than monolithic, with consideration given to the specific function a phone user engages in.

Illustrative of this notion, in study 2, we examined in the causal impact of mealtime food photography, which is one form of phone use that has come under scrutiny in both clinical and lay circles. Relative to nonfood photography, food photography disrupted the increase in food consumption that is typically observed across a day [38-40]. Thus, when participants took photographs of their food during a meal, they failed to discriminate between breakfast, lunch, and dinner portion sizes. This pattern is reminiscent of other situational factors (eg, multitasking, eating with peers, and use of digital screens) that distract an individual from eating, such that portion sizes become less driven by internal biological cues (eg, circadian rhythms, homeostatic signals) than by the external environment [46-48]. In turn, this form of external eating may predispose an individual to weight gain over time [47,49,50]. From a clinical perspective, our findings thus support the exploration of mealtime environments, including mealtime phone use habits, when addressing issues of abnormal weight and eating behavior. Nonetheless, we emphasize the preliminary nature of our findings, and the need for further replication.

More broadly, our finding that mealtime phone use is overwhelmingly social highlights another mechanism through which mobile phones may influence eating. This is particularly notable given that participants reported using more nonsocial functions outside the meal context, and because this manner of phone use distinguishes mobile phones from traditional digital screens (where little social interaction occurs). In previous research, one of the most robust determinants of food intake has been found to be the company one eats with: compared to eating alone, eating with others has been repeatedly found to increase consumption, a phenomenon termed *social facilitation* [51-53]. If facilitation can occur with virtual company [54-56], then social interactions during mealtime phone use (eg, through sending text messages) may likewise increase portion sizes [26]. Further research is needed to examine how these social forms of phone use may influence eating behaviors.

Finally, although the discussion has focused on what a phone user does, we also examined how much an individual engages in mealtime phone use (study 1). Here, we found no evidence that frequency altered the risk of obesity: BMIs, routine physical activities, and habitual eating behaviors did not differ significantly between participants who recorded high versus low engagement in mealtime phone use.

### Limitations

In presenting these various findings, we note several limitations in both variable and participant selection. First, given the current guidelines on mealtime screen use [12-14], we focused only on phone use within this context. Accordingly, we could not assess the nature and impact of general phone use across the day. Second, we chose to study young adults (aged 18-30 years), the age group most likely to own and be dependent on smartphones [57]. Although this maximized our ability to capture mealtime phone use, further research will need to examine whether our findings generalize to other age groups. Finally, we only collected data on participants’ 3 main meals and excluded snacks consumed outside of these times. As meal times are fairly stable, this strategy increased our chance of capturing when participants had eaten (study 1) or were about to eat (study 2). However, future studies can extend our work by exploring the impact of phone use on food intake across the day.

### Conclusions

For the first time, we characterized the nature of mealtime phone use and its implications for eating behaviors. By using experience sampling, we tracked a large number of meals in situ (1780 meals across 2 studies) and captured phone use as it occurred in its natural environment [58]. Through this approach, we circumvented recollection failures associated with self-reported phone use data [34] and documented the wide variety of forms through which individuals use their phones during a meal. Further, our preliminary findings suggest that certain types of phone use behaviors may influence food intake. Given the relevance of this topic for clinical practice and guidelines, our findings underscore the importance of follow-up research and the need to move beyond the broad notion of “screen time” to examine how individual phone functions contribute to an obesogenic environment.

## Appendix

Multimedia Appendix 1 Flow diagram of participant selection and dropout.

Multimedia Appendix 2 Demographic, baseline mealtime and phone use, and experience sampling questionnaires.

Multimedia Appendix 3 Validation of measures.

Multimedia Appendix 4 Sample images in the food and nonfood photography conditions.

Multimedia Appendix 5 Scatterplot of mealtime phone use frequency against number of meals captured.

Multimedia Appendix 6 Multilevel model of food eaten and enjoyment of food (study 2).

## Abbreviations

DEBQ: Dutch Eating Behavior Questionnaire
