# Supplementary figures and images for: Meal-time Smartphone Use in an Obesogenic Environment: Two Longitudinal Observational Studies
Source: JMIR Mhealth Uhealth. 2021 May 6;9(5):e22929. doi: 10.2196/22929 (PMC8138713; doi:10.2196/22929)

## Appendix A

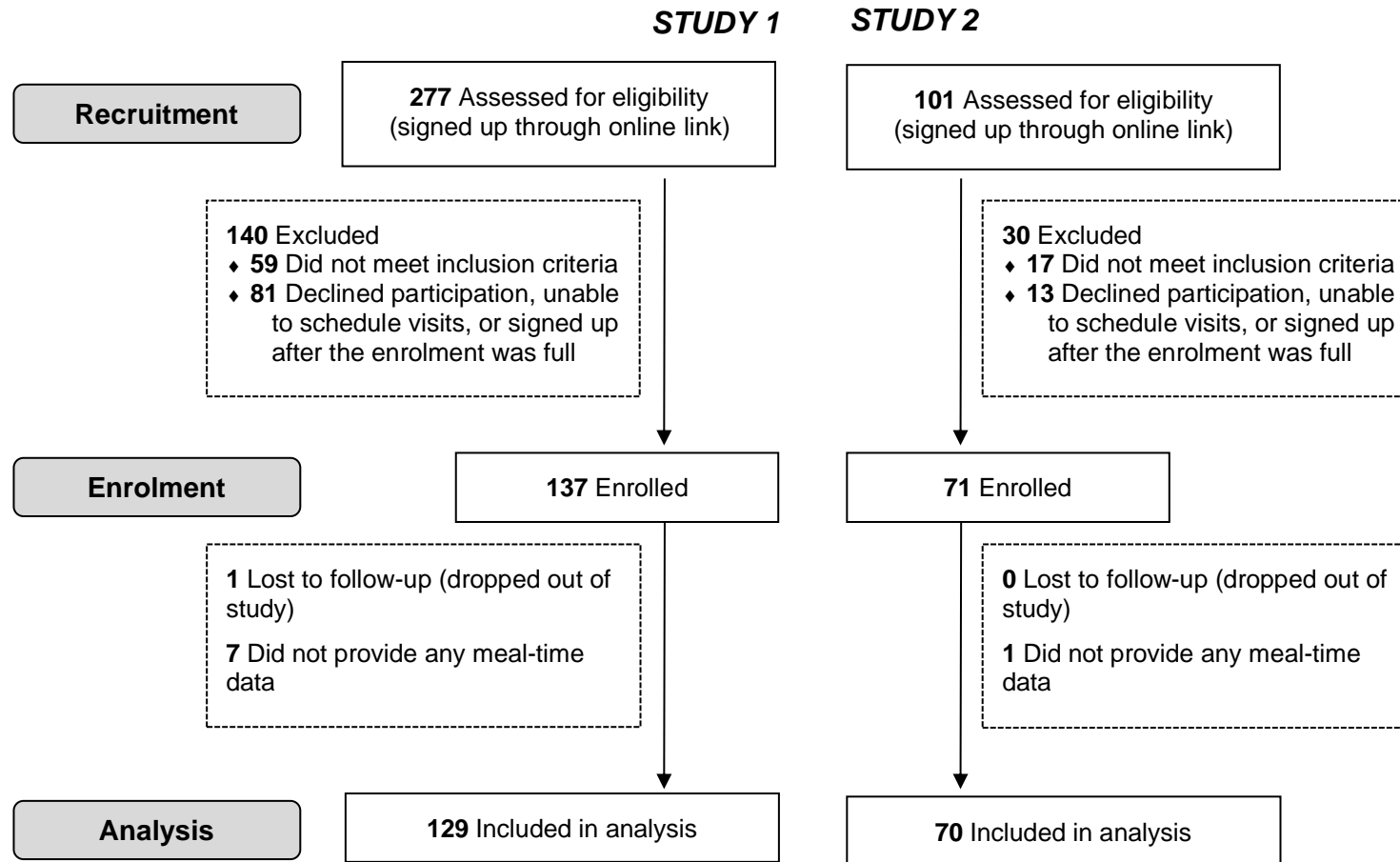

Figure S1. Flow diagram of participant selection and drop-out.

Supplement: Multimedia Appendix 1 [file mhealth_v9i5e22929_app1.pdf]
