# Supplementary material for: Meal-time Smartphone Use in an Obesogenic Environment: Two Longitudinal Observational Studies
Source: JMIR Mhealth Uhealth. 2021 May 6;9(5):e22929. doi: 10.2196/22929 (PMC8138713; doi:10.2196/22929)
Supplement: Multimedia Appendix 2 [file mhealth_v9i5e22929_app2.pdf]

Appendix B

Demographic questionnaire

1. What is your gender?
2. What is your age?
3. What is your current height?
4. What is your current weight?
5. What is your ethnicity?
6. What is your first language?
7. Do you have Instagram installed in your phone and frequently use the app?
8. How many followers do you have on Instagram?
9. How many accounts do you follow on Instagram?
10. On average, how many photos do you post on Instagram per week?
11. Which of the following categories do your photos on Instagram fall under?
    - Selfies
    - Friends & family
    - Food
    - Scenery
    - Everyday objects
    - Conceptual/ abstract
12. Are you dieting or in any way restricting the food you consume?
13. Do you have any food allergies?
14. How many times a week do you engage in vigorous physical activity long enough to work up a sweat?
    - 0-2 times/ week
    - 3-4 times/ week
    - 5-7 times/ week
15. Do you smoke?
16. Do you consume recreational drugs?
17. Do you have any chronic medical illnesses?
18. Do you have a history of any psychiatric condition?
19. Do you have a history of any eating disorder?

Baseline mealtime and phone use questionnaire

1. On average, what time do you eat breakfast?
2. On average, what time do you eat lunch?
3. On average, what time do you eat dinner?
4. What is the brand of the mobile phone you are currently using?
5. On a regular day, how likely are you to use your smartphone for these phone functions? (1 – Very unlikely, 5 – Very likely)
    - Talking on the phone
    - Messaging (e.g. SMS/ WhatsApp/ Telegram)
    - Emailing
    - Taking photos/ videos
    - Sharing photos/videos (e.g. Instagram/ Snapchat)
    - Watching videos
    - Playing games
    - Listening to music
    - Browsing websites
    - Others: __________
6. On a regular day, how likely are you to use your smartphone in these situations? (1 – Very unlikely, 5 – Very likely)
    - In bed when you wake up
    - In bed before you fall asleep
    - In the middle of the night (e.g. when your sleep is interrupted)
    - During meals
    - In the toilet
    - During class time
    - Idle time (e.g. while waiting for something or someone)
    - While waiting in line
    - During commute

Experience sampling questionnaire (Study 1)

1. Have you eaten in the past 15 minutes?
2. What did you eat?
3. How much did you enjoy your food? (1 – Not at all, 7 – Very much)
4. How much did you eat? (1 – Less than normal, 7 – More than normal)
5. Did you use your phone while eating?
6. What functions on your phone did you use?

Experience sampling questionnaire (Study 2)

1. Will you be eating in the next 15 minutes?

[If participants respond 'yes' to the above, they are randomly assigned to receive ***one*** *of the following three messages]*

A. Please take 5 photographs of your food and post the best one on Instagram
B. Please take 5 photographs of your surroundings (e.g. furniture, decorations) and post the best one on Instagram
C. Please refrain from using your phone while eating

[30 minutes later]

2. Did you post the photo on Instagram? If yes, please reply with a screenshot of the post. *(Question not displayed if participants were assigned to message C)*
3. What did you eat?
4. How much did you enjoy your food? (1 – Not at all, 7 – Very much)
5. How much did you eat? (1 – Less than normal, 7 – More than normal)
