# Supplementary material for: Meal-time Smartphone Use in an Obesogenic Environment: Two Longitudinal Observational Studies
Source: JMIR Mhealth Uhealth. 2021 May 6;9(5):e22929. doi: 10.2196/22929 (PMC8138713; doi:10.2196/22929)
Supplement: Multimedia Appendix 3 [file mhealth_v9i5e22929_app3.pdf]

**Appendix C**

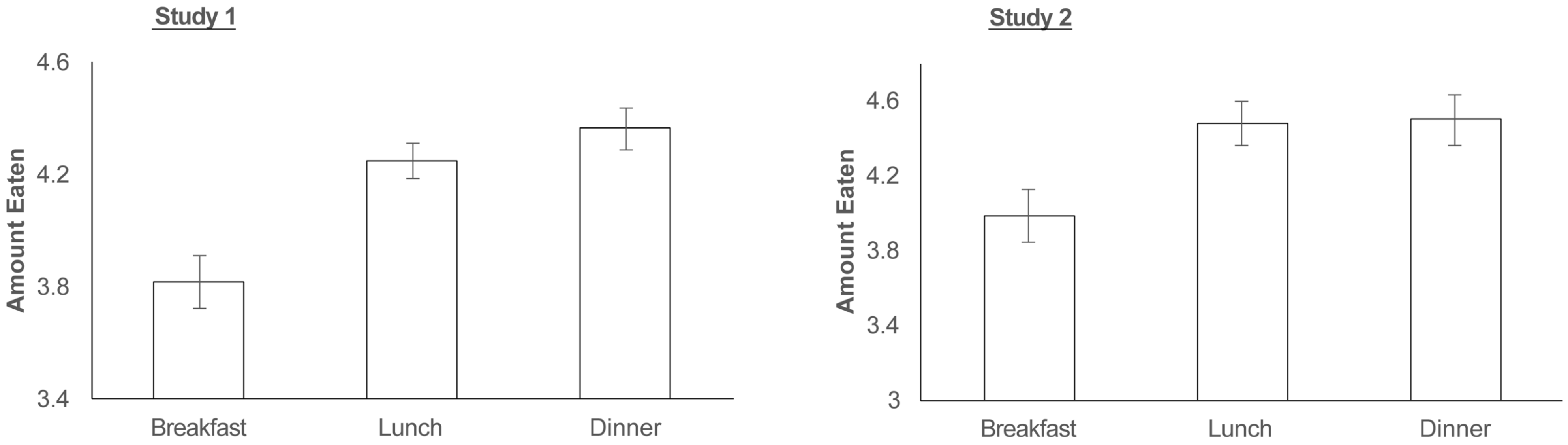


*Figure S2.* Mean amount eaten in Studies 1 (left) and 2 (right), plotted as a function of meal type (breakfast, lunch, or dinner). Vertical lines represent 1 standard error of the mean.

To validate the relative measurement approach of Study 2, we examined whether participants' reports of how much they had eaten would replicate the oft-reported finding that food intake increases across the day (amongst free-living individuals). To this end, we ran a linear mixed-effects model involving meal type (Results: Impact of Meal-Time Phone Use Patterns on Eating Behaviours (Study 2): Table 2). As shown in Figure S2, food intake did indeed increase across the day (main effect of meal type: $t(276.66) = 3.12$, $P = 0.002$).

As further validation, we examined corresponding data from Study 1 where the same measure had been included as pilot data for Study 2. Factoring the key variables of the design, we selected all instances where a meal was recorded (130 participants, 1140 observations), and coded whether participants used their phones during the meal (0 = 'no', 1 = 'yes'). This variable was grand-mean centred, and apportioned into between- and within-subjects components. Finally, we ran a linear mixed-effects model with the amount eaten as the outcome variable, and time (centred and divided by 3 to put the unit in days), phone use (between- and within-subject variables), meal type (breakfast, lunch, or dinner), and the interaction between each phone use variable and meal type entered as fixed effects. Random intercepts and slopes (for within-subject phone use) accounted for correlated data due to repeated measures. Again, as shown in Figure S2, food intake increased across the day (main effect of meal type: $t(990.39) = 5.39$, $P < 0.001$). Thus, across two independent samples, our experience sampling measure was sensitive to changes in portion sizes across the day.
