# Supplementary material for: Meal-time Smartphone Use in an Obesogenic Environment: Two Longitudinal Observational Studies
Source: JMIR Mhealth Uhealth. 2021 May 6;9(5):e22929. doi: 10.2196/22929 (PMC8138713; doi:10.2196/22929)
Supplement: Multimedia Appendix 4 [file mhealth_v9i5e22929_app4.pdf]

Appendix D

Food Photography

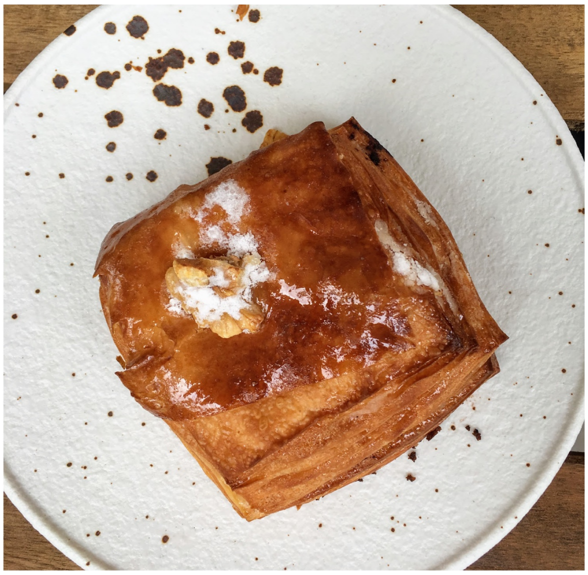

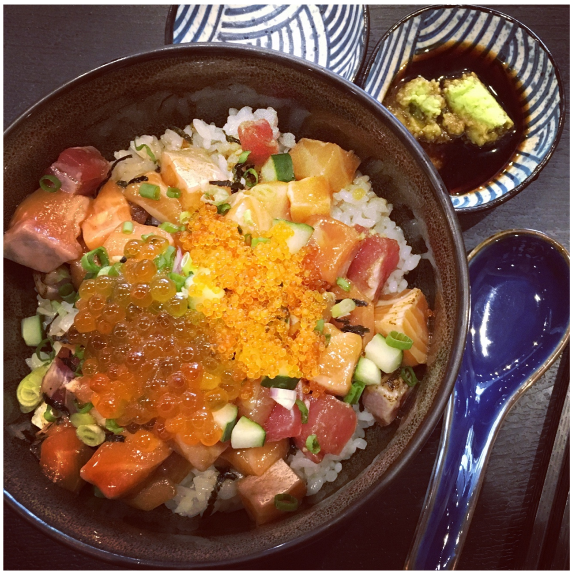

Non-Food Photography

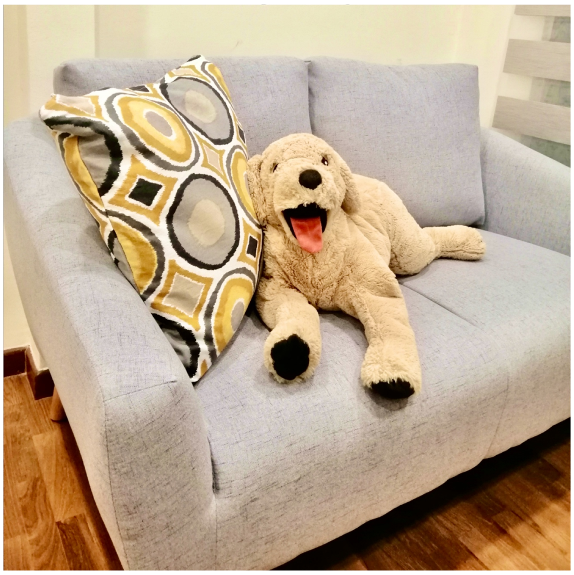

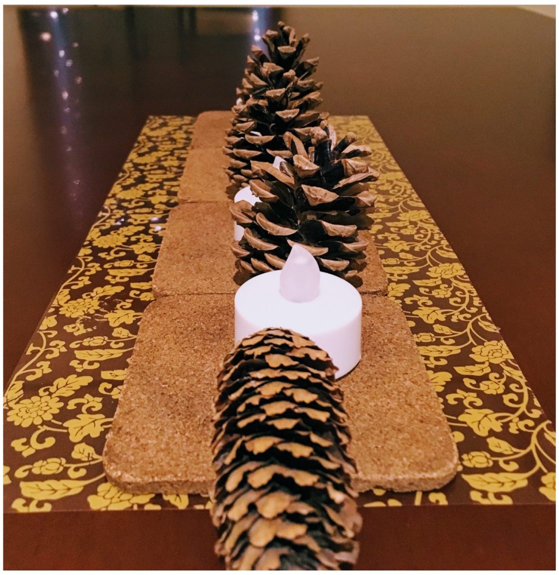

Figure S3. Sample images in the food and non-food photography conditions (Study 2).
