# Supplementary material for: Meal-time Smartphone Use in an Obesogenic Environment: Two Longitudinal Observational Studies
Source: JMIR Mhealth Uhealth. 2021 May 6;9(5):e22929. doi: 10.2196/22929 (PMC8138713; doi:10.2196/22929)
Supplement: Multimedia Appendix 5 [file mhealth_v9i5e22929_app5.pdf]

Appendix E

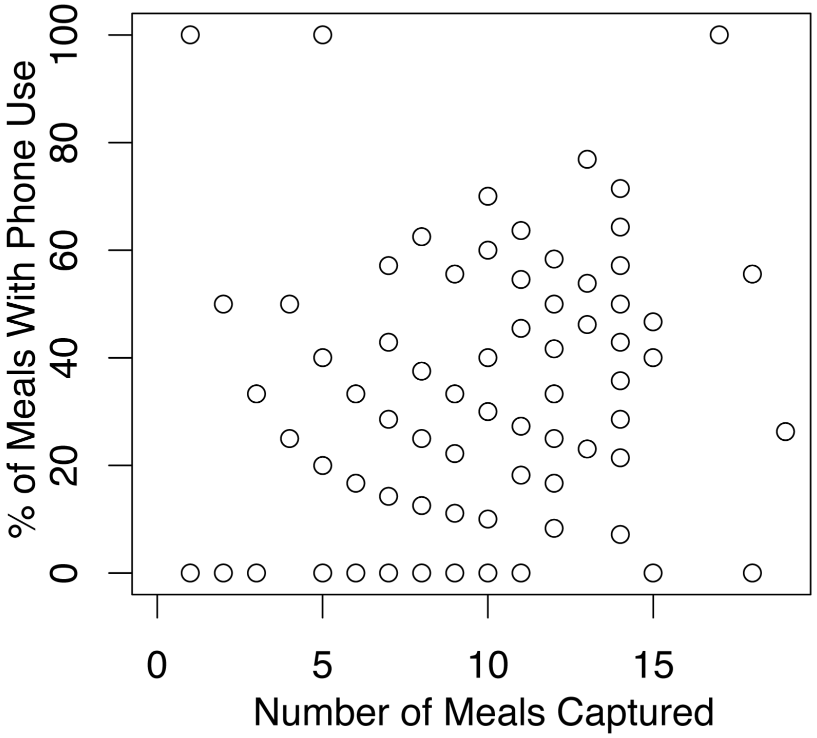

Figure S4. Scatterplot of meal-time phone use frequency against the number of meals captured.

When describing individual differences in meal-time phone use, one concern is that the measure of frequency may simply be an artefact of the number of meals recorded (through the experience sampling procedure). If this were the case, one would expect higher or more varied frequencies with fewer meals captured. However, Figure S4 does not suggest this trend, with chronic meal-time phone users observed regardless of the number of meals recorded.
