# Supplementary material for: Meal-time Smartphone Use in an Obesogenic Environment: Two Longitudinal Observational Studies
Source: JMIR Mhealth Uhealth. 2021 May 6;9(5):e22929. doi: 10.2196/22929 (PMC8138713; doi:10.2196/22929)
Supplement: Multimedia Appendix 6 [file mhealth_v9i5e22929_app6.pdf]

Appendix F

Parameter estimates for the multi-level model of amount of food eaten (Model 1) and enjoyment of food (Model 2) as a function of participants' meal-time phone use condition.

	Estimate	SE	*t*	*P*	CI_{95} lower	CI_{95} upper
**(Model 1) Outcome: Amount of food eaten**						
Fixed effects						
Intercept	3.85	0.18	21.18	<0.001	3.49	4.20
Time (per 3 meals)	-0.01	0.03	-0.17	0.86	-0.07	0.06
Phone use (phone use vs. none)	-0.27	0.26	-1.02	0.31	-0.78	0.25
Photography (food vs. non-food)	0.49	0.20	2.43	0.02	0.09	0.88
Meal type	0.24	0.08	3.12	0.002	0.09	0.39
Phone use x meal type	0.13	0.11	1.15	0.25	-0.09	0.35
Photography x meal type	-0.23	0.09	-2.61	0.009	-0.40	-0.06
Random effects						
Level 2 (between-person)						
Intercept	0.25	0.09	2.79	0.005	0.12	0.50
Level 1 (within-person)						
Residual	1.08	0.09	11.77	<0.001	0.91	1.28
Autocorrelation	-0.04	0.10	-0.42	0.67	-0.24	0.15
**(Model 2) Outcome: Enjoyment of food**						
Fixed effects						
Intercept	5.00	0.18	28.21	<0.001	4.65	5.34
Time (per 3 meals)	-0.02	0.03	-0.58	0.56	-0.09	0.05
Phone use (phone use vs. none)	0.08	0.26	0.30	0.76	-0.44	0.60
Phone use (food vs. non-food)	-0.02	0.20	-0.11	0.91	-0.42	0.38
Meal type	0.17	0.08	2.20	0.03	0.02	0.32
Phone use x meal type	0.08	0.11	0.71	0.48	-0.14	0.31
Photography x meal type	0.03	0.09	0.36	0.72	-0.14	0.21
Random effects						
Level 2 (between-person)						
Intercept	0.09	0.06	1.37	0.17	0.02	0.36
Level 1 (within-person)						
Residual	1.18	0.10	11.68	<0.001	1.00	1.40
Autocorrelation	0.03	0.08	0.42	0.68	-0.13	0.19
